# Endocrine Mechanisms Regulating Post-Diapause Development in the Cabbage Armyworm, *Mamestra brassicae*

**DOI:** 10.1371/journal.pone.0146619

**Published:** 2016-01-08

**Authors:** Nobuto Yamada, Naoki Okamoto, Hiroshi Kataoka, Akira Mizoguchi

**Affiliations:** 1 Division of Biological Science, Graduate School of Science, Nagoya University, Nagoya, Japan; 2 Department of Integrated Biosciences, Graduate School of Frontier Sciences, The University of Tokyo, Kashiwa, Chiba, Japan; University of Würzburg, GERMANY

## Abstract

Diapause, a programmed developmental arrest at a specific stage, is common in insects and is regulated by hormones. It is well established that in pupal diapause, cessation of ecdysteroid secretion from the prothoracic glands (PGs) after pupal ecdysis leads to diapause initiation, while resumption of its secretion induces post-diapause development. However, what regulates the activity of the glands is poorly understood, especially for the glands of diapause-terminated pupae. In the present study, we investigate the mechanisms by which post-diapause development is regulated in the cabbage armyworm *Mamestra brassicae*. We demonstrate that the brain is necessary for the initiation of post-diapause development and that the factor in the brain responsible for the activation of the PGs is the prothoracicotropic hormone (PTTH). Further, through measuring the hemolymph PTTH titers by time-resolved fluoroimmunoassay, we show that PTTH is actually released into the hemolymph prior to the activation of the PGs. Although its peak titer is much lower than expected, this low concentration of PTTH is most likely still effective to activate the PGs of post-diapause pupae, because the responsiveness to PTTH of the glands at this stage is very high compared to that of nondiapause pupal PGs. These results strongly suggest that in *M*. *brassicae*, PTTH serves as a trigger to initiate pupa-adult development after diapause termination by stimulating the PGs to secrete ecdysteroid.

## Introduction

Insects have evolved various mechanisms of adaptation to the environmental changes of their habitat. For example, many species enter diapause, a programmed developmental arrest, to survive a cold or dry season unsuitable for their life. Insects enter diapause at various developmental stages, i.e., egg, larva, pupa, and adult, but the stage at which diapause occurs is fixed in each species. In many cases, diapause is induced by seasonal changes in day length and terminates after the experience of low temperature for several months, although some insects use other environmental cues for the regulation of diapause (for reviews, see [1, 2]).

It is widely known that hormones are involved in the regulation of diapause [3]. Historically, the oldest and most famous study on the endocrine regulation of diapause is that of pupal diapause in *Hyalophora cecropia* by Carroll Williams in the mid 20^th^ century [4–6]. This study was actually aimed at determining the roles of the brain and the prothoracic glands (PGs) in the regulation of metamorphosis (pupa-adult development). Using diapause and post-diapause pupae effectively, he demonstrated that metamorphosis is induced by a molting hormone secreted by the prothoracic glands (PGs) and these glands are activated by a hormone secreted by the brain, which he referred to a “brain hormone”. Later, this relation of the brain and the PG was confirmed in many insects and is now recognized as a central axis of the endocrine system regulating insect development. Since diapausing pupae were used in this study, the results of his experiments also provided important suggestions about the mechanism of pupal diapause. Williams suggested in his paper that the cessation of secretion of the brain hormone after pupal ecdysis and the resultant PG inactivation lead to pupal diapause, while resumption of its secretion terminates diapause, leading to adult development [6]. Later, the cessation and resumption of secretion of the PG hormone now termed ecdysteroids in the initiation and termination of pupal diapause, respectively, were confirmed in many insects through measuring hemolymph titers of ecdysteroids by radioimmunoassay [7–10]. Thus, the roles of ecdysteroids in the regulation of pupal diapause have been well established.

However, since the structure of the brain hormone (now called prothoracicotropic hormone or PTTH, for its action on the PG) was unknown for a long time, the studies on the role of PTTH in the regulation of pupal diapause were limited. The structure of PTTH was determined partially in 1987 and completely in 1990 in *Bombyx mori* [11, 12]. *Bombyx* PTTH is a homodimer of 109-amino acid subunits [13]. Since then, the PTTH gene has been cloned in many species [14]. In all the species examined, PTTH is produced by two pairs of neurosecretory cells in the brain and axonally transported to the corpora allata (CA), from which PTTH is released into the hemolymph, as demonstrated by *in situ* hybridization and/or immunocytochemistry [14–16].

Recently, we have started studying the endocrine mechanisms regulating pupal diapause in the cabbage armyworm *Mamestra brassicae*, with a special interest in the roles of PTTH in the regulation of diapause. In *M*. *brassicae*, pupal diapause is induced under short-day conditions, as in many other insects. Diapausing pupae do not initiate adult development for months if they are maintained under warm conditions, but they start adult development (post-diapause development) if chilled at 4°C for 2 months and then transferred to warm conditions. In our previous reports, we demonstrated that the cessation of PTTH secretion after pupal ecdysis is the critical determinant of diapause initiation [16]. In the present study, we investigate the endocrine mechanisms regulating the initiation of post-diapause development. In contrast to the established role of ecdysteroids in the initiation of adult development, the involvement of PTTH in the activation of the PGs at this stage has been a matter of debate, although many researchers have speculated that PTTH is responsible for this process, because in some insects the brain becomes unnecessary for adult development well before diapause termination [1]. Here we show that PTTH plays a key role in the initiation of post-diapause adult development in *M*. *brassicae*. We also demonstrate that PTTH secretion in the post-diapause pupae is less significant than in nondiapause pupae but is still effective to activate the PGs due to a very high sensitivity of the glands to PTTH at this stage.

## Materials and Methods

### Animals

Eggs of *M*. *brassicae* were obtained from a laboratory colony maintained at the National Institute of Agrobiological Sciences, Japan. Larvae were reared on an artificial diet ‘‘Insecta LFS” (Nihon Nosan Kogyo, Yokohama, Japan) at 25°C under a 14-h light/10-h dark photoperiod (long-day conditions) or at 23°C under a 10-h light/14-h dark photoperiod (short-day conditions). The animals under the long-day and short-day conditions entered pupal diapause at rates of 0% and 100%, respectively. Diapausing pupae were maintained at 25°C for six weeks after pupation and then chilled at 4°C for eight weeks in most cases and for a shorter period in a specific experiment. The chilled pupae were transferred to an incubator set at 25°C (hereafter called warm conditions) to allow resumption of development. These pupae were consistently exposed to a 12-h light/12-h dark photoperiod.

### Antibodies

Anti-*Mab*PTTH mouse monoclonal and rabbit polyclonal antibodies and anti-ecdysone rabbit antiserum were the same as those used in our previous study [16]. Horseradish peroxidase-labeled anti-rabbit IgG secondary antibody and biotin-labeled anti-rabbit IgG secondary antibody were purchased from Boehringer Mannheim and Amersham Biosciences, respectively.

### Collection and processing of hemolymph for determination of ecdysteroid and PTTH titers

Hemolymph was sampled at 12 h intervals, starting immediately after the transfer of the animals from the refrigerator to the warm conditions. For ecdysteroid determination the hemolymph was processed as described previously [16]. For PTTH determination, the hemolymph (200 μl) was diluted 1:3 with 50 mM Tris-buffered saline, pH 7.6 (TBS) and heated at 70°C for 5 min. After cooling and centrifugation of the heated sample, 6 μl of 10% trifluoroacetic acid (TFA) was added to the supernatant. PTTH in the sample was extracted by solid-phase extraction using a Sep-Pak C8 cartridge (Waters) and eluted with 40% acetonitrile containing 0.1% TFA. The eluate was freeze-dried, and dissolved in 200 μl of TBS containing 0.1% bovine serum albumin.

### Determination of ecdysteroid titers in the hemolymph by enzyme-linked immunosorbent assay (ELISA)

The wells of EIA plates (Costar, 3590) were incubated with 50 μl of 20-hydroxyecdysone (20E)-ovalbumin conjugate overnight at 4°C, as previously described [16], followed by blocking with 120 μl of ‘BLOCK-ACE’ (Yukijirushi Megumilk, Hokkaido, Japan) for 1 h at 25°C. After washing the wells as previously described [16], 50 μl of serially diluted 20E or test samples were distributed to the wells, followed by the addition of 50 μl of 1:100,000 diluted anti-ecdysone rabbit antiserum. After overnight incubation at 4°C, the wells were washed, and incubated with 1:10,000 diluted horseradish peroxidase-labeled secondary antibody. After a 1 h incubation at 25°C, the wells were washed and developed with *o*-phenylenediamine. The absorbance at 492 nm was measured using a 96-well plate reader, Multiskan FC microplate photometer (Thermo Fisher Scientific).

### Determination of PTTH titers in the hemolymph by time-resolved fluoroimmunoassay (TR-FIA)

TR-FIA for PTTH was performed as described previously [16].

### Brain ablation, allatectomy and implantation

Prior to surgery, pupae were anesthetized by submersion in water for 1 h. Then the tip of the head was cut open, and the brain or brain-corpora cardiaca-corpora allata complex (Br-CC-CA) was removed. When allatectomy was conducted, the CC was also removed together with the CA, because the two organs are closely associated and inseparable.

To prepare the brains for implantation, the tip of the head was cut off, and the brain was collected in insect saline [17]. The brain was implanted into the debrained pupa immediately after the removal of the host brain. The wound was sealed with paraffin. A sham-operation was performed by cutting the head open but keeping the Br-CC-CA complex untouched.

### Preparation and injection of crude PTTH

Brain extract (crude PTTH) was prepared as described previously [16]. The amount of PTTH contained in the brain extract was determined by TR-FIA. Since we have no pure standard PTTH, the amount of PTTH was expressed in a relative amount, with 1 unit corresponding to the PTTH amount contained in a brain of day-1 nondiapause-destined wandering larva. To remove PTTH from the brain extract, immunoprecipitation was performed as described previously [16]

Prior to injection, pupae were anesthetized by submersion in water for 1 h. Ten microliters of the brain extract (2 units of PTTH equivalents) was then injected into the tip of the head. The incisions were sealed with melted paraffin.

### *In vitro* culture of the PG

*In vitro* culture of the PGs was performed as described [16] with the following modifications: the preincubation time was 1 h, and the volume of the medium was 50 μl.

### Affinity purification of PTTH from the brain extract

Anti-*Mab*PTTH rabbit antibody was bound to a Hitrap affinity column (Pharmacia Biotech) according to the manufacturer’s instructions.

Two milliliters of the brain extract was applied onto the column. PTTH was eluted with 2 ml of 0.2M Glycine-HCl (pH 2.5). The affinity-purified PTTH was extracted from the eluate using a Sep-Pac C8 cartridge: The eluate was mixed with 20 μl of 10% TFA and applied onto the cartridge, and the bound PTTH was eluted with 40% acetonitrile followed by freeze-drying.

### Preparation of PTTH-containing agarose gel and its implantation into debrained pupae

Four percent agarose gel was prepared using agar powder (Wako) and 0.9% NaCl solution. The gel was cut into small pieces (1mm x 1mm x 1mm) and then soaked in PTTH solution (0.5 units/μl; 0.9% NaCl) for 2 days at 4°C. The gel was implanted into the head of the debrained pupa immediately after removal of the brain.

### Molecular Cloning of *MabTorso*

Total RNA was extracted from 10 PGs of day-0 sixth instar larvae using RNeasy Mini Kit (QIAGEN). The reverse transcription to obtain cDNA was performed using PrimeScript Reverse Transcriptase (TaKaRa). The following degenerated primers were designed from consensus sequences of previously determined *Torso* cDNA sequences for *B*. *mori* and *Drosophila melanogaster*.

*MabTorso* cloning forward: 5’- CGGCGAAGGAGCCTT(C/T)GGC(G/A)T(C/G)GTGCG -3’

*MabTorso* cloning reverse: 5’- CAC(A/G)CC(A/G)AAGGACCAAACATC(G/A)CTCTG -3’

PCR was performed under the following conditions: 94°C for 2min and 35 cycles of 94°C for 30 sec, 60°C for 30 sec, and 72°C for 40sec, with a final extension step of 7 min at 72°C. The PCR products were purified using the Wizard SV Gel and PCR Clean-Up System (Promega), cloned into a pGEM-T Easy vector (Promega) and sequenced. The entire sequence of *MabTorso* was determined by 5’ and 3’ RACE using the SMARTer RACE cDNA Amplification Kit (Clontech) and Advantage 2 Polymerase Mix (Clontech). The products of the 5’ and 3’ RACE were sequenced, and the obtained sequence and its deduced amino acid sequence were analyzed and aligned using GENETIX-MAC ver. 13.0.6 (GENETYX). The primers used were as follows:

*MabTorso* 5’RACE GSP1 reverse-1: 5’- GCCTCCTGCCGGTGCAGCATCCTACC -3’

*MabTorso* 3’RACE GSP2 forward-1: 5’- AAGGGCCACGCTCGCGCCGCACGG -3’

*MabTorso* 3’RACE Nested forward-2: 5’- TCATCGTCGCAGAGTACTGTAGC -3’

*MabTorso* 3’RACE forward-3: 5’- AGGAGATAGGACGTTGAAGATAGCGG -3’

*MabTorso* 3’RACE forward-4: 5’- CCATCCTCGCGAAAGACCGACCTT -3’

*MabTorso* 3’RACE forward-5: 5’- AGCGGGTCAGCCAAAATGGTCAGATC -3’

*MabTorso* 3’RACE forward-6: 5’- TGCACTACTTGCTGTGTTATGTGTCTG -3’

*MabTorso* 3’RACE forward-7: 5’- TCAGCTGAATCCGGTTTGACTGGAAG -3’

*MabTorso* 5’RACE reverse-2: 5’- GCCTCCTGCCGGTGCAGCATCCTACC -3’

*MabTorso* 5’RACE reverse-3: 5’- TCCCATCGGTCTTCTGTTTCGCTC -3’

*MabTorso* 5’RACE reverse-4: 5’- TTCAGGTGGCTGGTTCCGAACATG -3’

*MabTorso* 5’RACE reverse-5: 5’- TTTCGTCACTGTCCGTCGGTTTTCC -3’

The nucleotide sequence data of *MabTorso* is available in the DDBJ/EMBL/GenBank databases under the accession number LC076477.

### Quantitative RT-PCR

Total RNA was extracted from the larval or pupal brains and PGs using the RNeasy Mini Kit. The reverse transcription to obtain cDNA was described above. Quantitative RT-PCR (qRT-PCR) was performed as described previously [18]. For absolute quantification of mRNAs, serial dilutions of plasmids carrying cDNAs were used for standards. After the molar amounts were calculated, transcript levels of the genes were normalized with *RpL8* levels in the same samples. The *RpL8* primers and *PTTH* primers used for qRT-PCR were prepared as described previously [16]. The primers used for the qRT-PCR for *MabTorso* were as follows.

*MabTorso* expression forward: 5’- TCATCGTCGCAGAGTACTGTAGC -3’

*MabTorso* expression reverse: 5’- TCACGACCAGTTTAGAGCTCTCC -3’

### Statistical Analysis

One-way analysis of variance (ANOVA), Tukey-Kramer multiple comparison test, and Student t-test were used for statistical analysis.

## Results

### A period of chilling required for diapause termination

**Fig 1:**
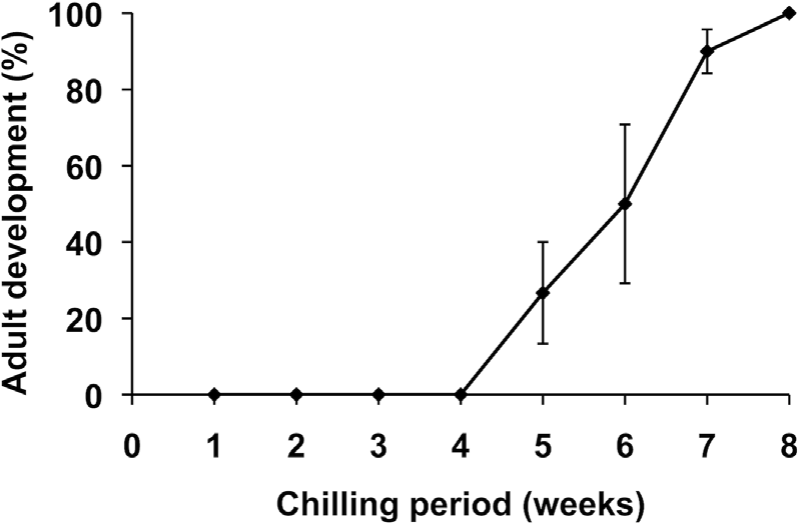
Duration of chilling period required for diapause termination. Diapausing pupae 6 weeks after pupal ecdysis were chilled at 4°C and a fraction (n = 10) of the chilled pupae transferred to an incubator set at 25°C every week. Adult development was judged by observing the wing vein apolysis from the epidermis within 10 days after warming, and the percentage of the animals that initiated adult development was calculated. The values shown are the means (±SEM) of three independent determinations.

If diapausing pupae are exposed to a low temperature for a sufficient period, they terminate diapause and initiate adult development under warm conditions. To determine the minimal chilling period necessary for diapause termination, a large batch of 6-week-old diapausing pupae was transferred to a refrigerator (4°C) and then a group of pupae was returned to the warm conditions (25°C) every week. No pupae initiated adult development when the chilling period was 4 weeks or less, but the percentage of pupae that terminated diapause increased linearly with the prolongation of the chilling period (Fig 1). When the pupae were chilled for 8 weeks or longer, all the pupae terminated diapause. Therefore, we used the pupae that had been chilled for 8 to 10 weeks in the following experiments. The 8-week-chilled, diapause-terminated, pupae never started adult development as long as they are maintained at 4°C (post-diapause quiescence).

### Changes in the hemolymph ecdysteroid titers during post-diapause development

**Fig 2:**
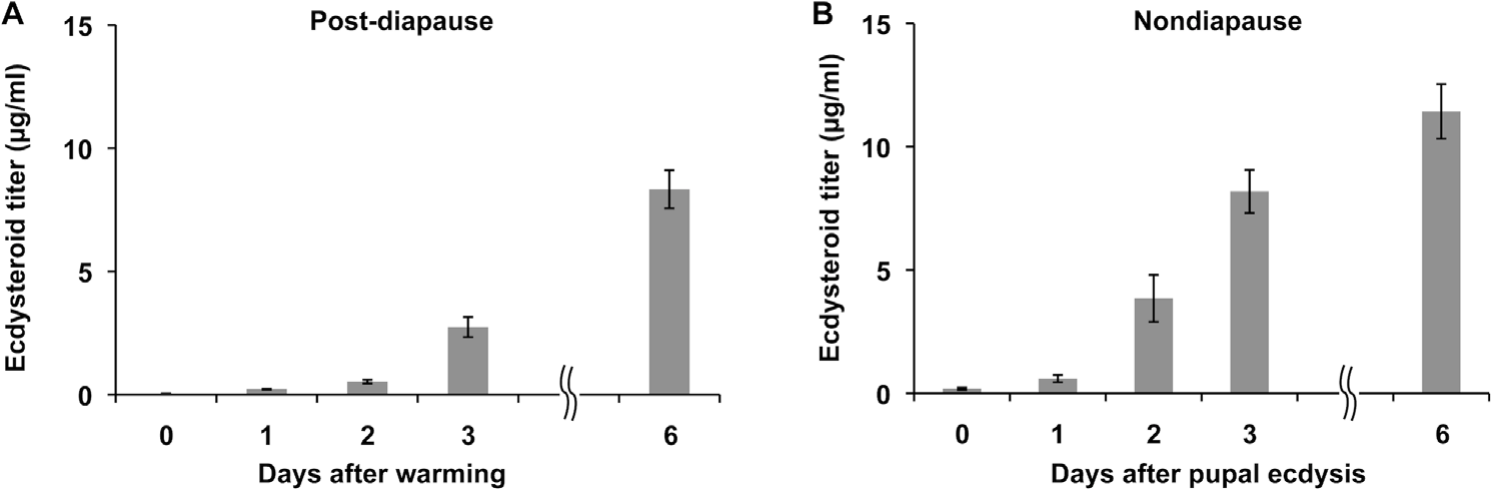
Changes in ecdysteroid titers in the hemolymph. Ecdysteroid titers in the hemolymph of post-diapause pupae (A) and nondiapause pupae (B) were determined by ELISA. The values are the means ±SEM (n = 6).

When diapause-terminated pupae are placed in warm conditions, the secretion of ecdysteroids from the PGs resumes in many insects [9,10]. To confirm that the same changes occur in *M*. *brassicae*, hemolymph ecdysteroid titers in post-diapause pupae were determined. For comparison, ecdysteroid titers in nondiapause pupae were also measured. The hemolymph ecdysteroid titers steadily increased after warming of the post-diapause pupae (Fig 2A and 2B) with a similar time course and magnitude to those in nondiapause pupae.

### Roles of the brain in post-diapause development

**Fig 3:**
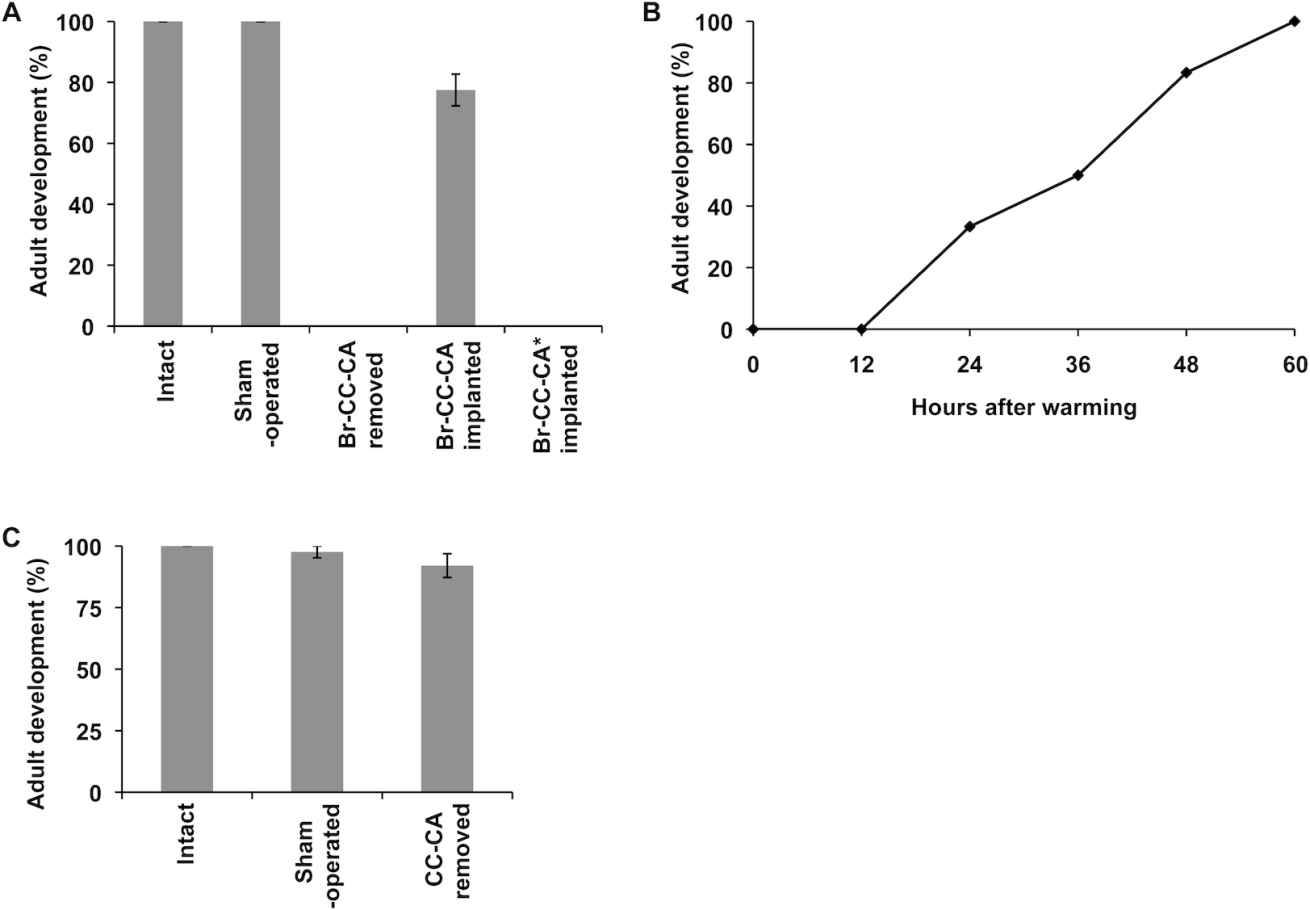
The role of the Br-CC-CA in post-diapause development. (A) The effects of brain removal and implantation on adult development of post-diapause pupae. Diapausing pupae were chilled for 8 weeks and the Br-CC-CA was removed immediately after warming. A Br-CC-CA collected from another previously chilled pupa was implanted into a Br-CC-CA-deficient pupa. As a positive control, pupae were only injured (sham-operated). For comparison, a Br-CC-CA collected from an unchilled pupa 5 weeks after pupation (*) was implanted into a Br-CC-CA-deficient pupa. Percentage values were determined with 6–12 pupae per operation. The values shown are the means (±SEM) of three independent determinations. (B) Time-dependent effects of brain removal on the development of post-diapause pupae. The Br-CC-CA was removed from post-diapause pupae at various times after warming (n = 12). (C) The effect of CC-CA removal (allatectomy) on adult development of post-diapause pupae. The CC-CA was removed immediately after warming. Percentage values were determined with 6–12 pupae per operation. The values shown are the means (±SEM) of three independent determinations.

The necessity of the brain in post-diapause development was examined. When the Br-CC-CA was removed immediately after warming, no pupae started adult development (Fig 3A). However, when the Br-CC-CA collected from pupae 12 h after warming was implanted into Br-CC-CA-deficient pupae, about 80% of the implanted pupae started development. These observations led us to conclude that the Br-CC-CA is necessary for post-diapause development and that the Br-CC-CA secretes some hormone that activates the PGs.

Next we determined when the Br-CC-CA is needed for the initiation of adult development. The Br-CC-CA was removed 0, 12, 24, 36, 48, or 60 h after warming. When the pupae were debrained at 0 h or 12 h after warming, the pupae did not resume development. However, when pupae were debrained at 24 h after warming, about 30% of them started adult development, and the percentage of pupae that resumed development increased proportional to the delay in the time of the operation, with brain removal at 60 h having no effect on adult development at all (Fig 3B). These results indicate that the Br-CC-CA is required for adult development for about 48 h after warming.

### The effects of brain extract injection

**Fig 4:**
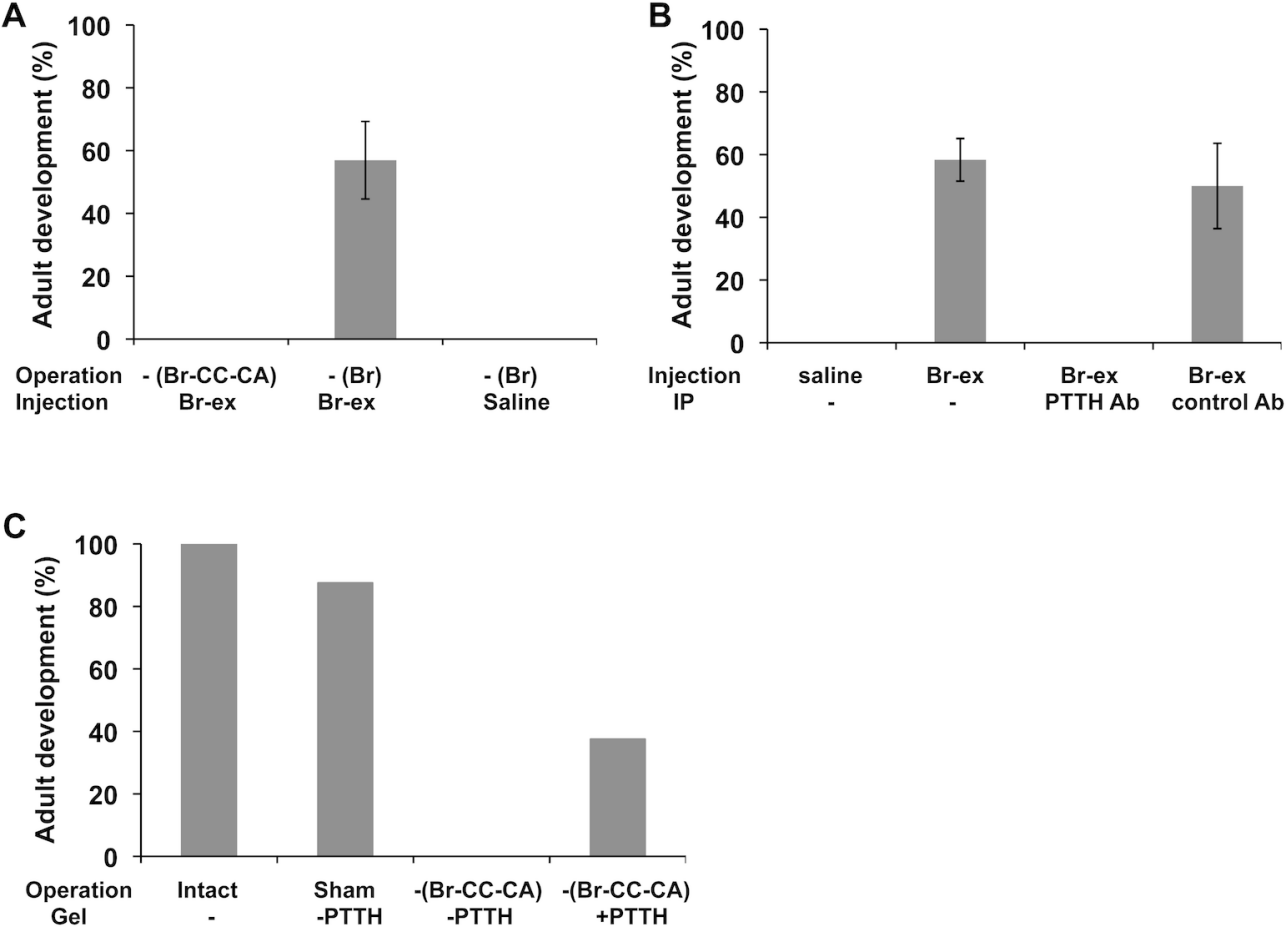
The role of PTTH in the initiation of adult development. (A) The effect of brain extract (Br-ex) injection. The Br-CC-CA or brain alone (Br) was removed from post-diapause pupae 12 h after warming and 12 h later, brain extract (2 units of PTTH equivalents) was injected. As a negative control, saline was injected into the brain-deficient pupae. (B) The effect of PTTH removal from the brain extract. The brains were removed from post-diapause pupae 12 h after warming, and the debrained pupae were injected with the brain extract pre-treated with an anti-*Mab*PTTH or control antibody. As a negative and a positive controls, saline and the same dose (3 units of PTTH equivalents) of the brain extract were injected, respectively (n = 6). The values shown are the means (± SEM) of three independent determinations. (C) The Effect of the implantation of PTTH-containing gel into the Br-CC-CA-deficient pupae. The Br-CC-CA was removed from post-diapause pupae immediately after warming, and the PTTH-containing gel (+PTTH) was implanted into the Br-CC-CA-deficient pupae (n = 8). Control pupae received the gel without PTTH (-PTTH).

The fact that the implantation of the Br-CC-CA into debrained pupae resulted in adult development strongly suggests that the brain secretes a hormone that induces adult development. If this is the case, the hormone should be contained in the brain extract. To test this prediction, we injected the brain extract into debrained pupae. The Br-CC-CA or the brain alone was removed from the diapausing pupa 12 h after warming, and 12 h later the brain extract containing 2 units of PTTH was injected. When the Br-CC-CA was removed, injection of the brain extract was unable to induce adult development. However, when only the brain was removed, the injection of the brain extract induced adult development, although the brain-deficient control animals never started development (Fig 4A). To identify this necessary brain hormone, we removed PTTH from the brain extract by immunoprecipitation, and then injected the extract into the debrained pupae in the same way. While control brain extract induced adult development, PTTH-absorbed brain extract failed to do so (Fig 4B). These results suggested that the hormone responsible for adult development is PTTH.

### The Effects of the implantation of PTTH-containing gel

The above results suggest that PTTH most likely triggers adult development of post-diapause *M*. *brassicae* pupae. However, the observation that the injection of PTTH (brain extract) to the Br-CC-CA-deficient pupae failed to induce adult development suggested the possibility that some other factor derived from the CC-CA is also responsible for the induction of adult development. Obviously, the involvement of juvenile hormone (JH), which is produced by the CA, in this process is eliminated, because when the CC-CA was removed immediately after warming, the operated pupae developed into adults without a significant delay (Fig 3C). Therefore, we hypothesized that the CC-CA serves as another source of PTTH, continuously releasing a small amount of PTTH to potentiate the PGs, because the CC-CA 12 h after warming contained almost the same amount of PTTH (0.10 U) as that in the brain (0.11 U). If a continuous action of PTTH on the PGs is critical for the activation of the glands at this stage, it is reasonable that a single injection of PTTH to the Br-CC-CA-deficient pupae could not activate the PGs. To examine this hypothesis, we developed a new method to continuously supply PTTH to pupae. Affinity-purified PTTH was loaded into an agarose gel, and a small piece of the gel was implanted into the Br-CC-CA-deficient pupae that were operated on immediately after warming. Approximately 40% of the operated pupae started adult development (Fig 4C), strongly supporting the hypothesis.

### Changes in hemolymph PTTH titers during post-diapause development

**Fig 5:**
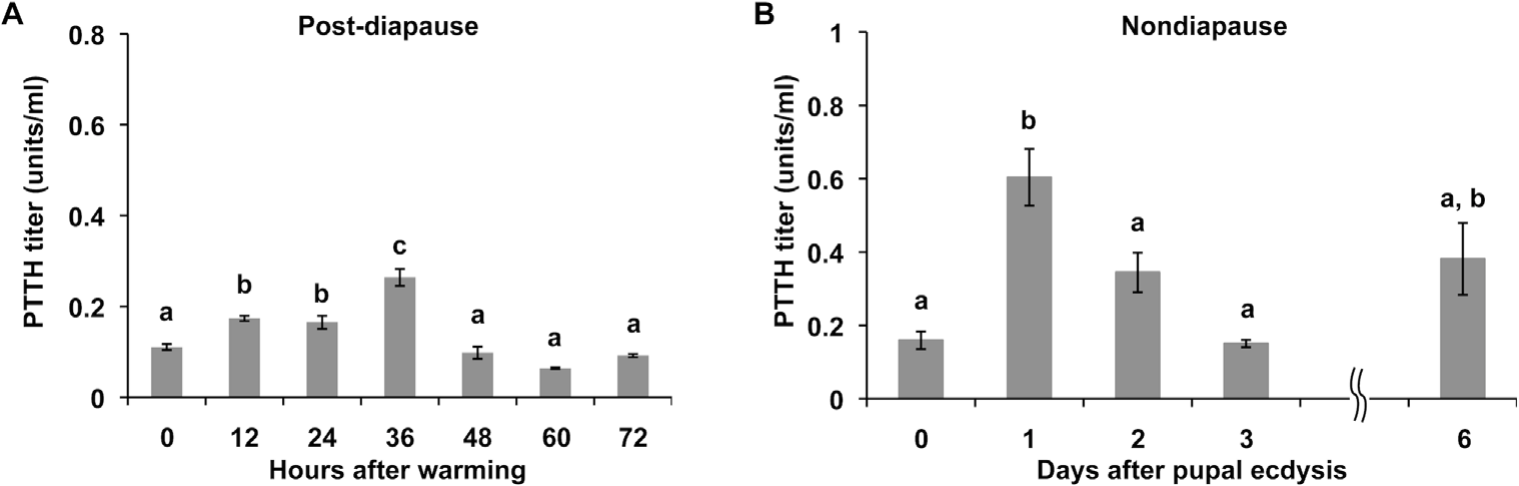
The changes in PTTH titers in the hemolymph. PTTH titers in the hemolymph of post-diapause pupae after warming (A) and nondiapause pupae after pupal ecdysis (B) were determined by TR-FIA. The values are the means ±SEM (n = 8). Different letters above the bars indicate a significant difference (ANOVA followed by Tukey-Kramer multiple-comparison test, p <0.05).

If PTTH triggers the initiation of adult development, the secretion of PTTH should be detected after the pupae are warmed. Therefore, the hemolymph PTTH titers after warming were determined by time-resolved fluoroimmunoassay (TR-FIA). The titer gradually increased until 36 h after warming and then gradually decreased (Fig 5A). This time course of PTTH titer change was consistent with the period of time during which Br-CC-CA was required for the initiation of adult development (Fig 3B). However, the peak titer of PTTH was only about one third as compared with that during early development of nondiapause pupae (Fig 5B).

### Developmental changes in PTTH gene expression and PTTH content in the brain

**Fig 6:**
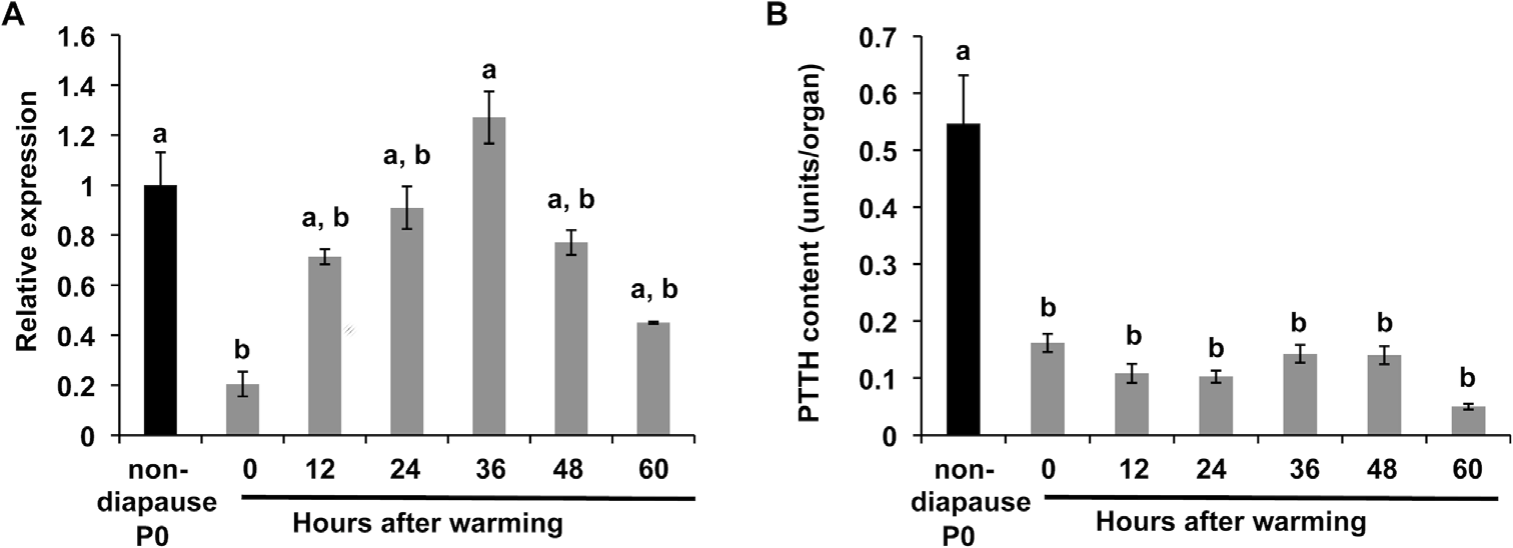
Developmental changes in *PTTH* gene expression and PTTH content in the brain. (A) *PTTH* gene expression in the brains of day-0 nondiapause pupae and post-diapause pupae at various times after warming was analyzed by qRT-PCR. The values shown are the means (± SEM) of three independent determinations. (B) PTTH content in the brains of day-0 nondiapause pupae and post-diapause pupae at various times after warming was determined by TR-FIA. The values are the means ±SEM (n = 6). Different letters above the bars indicate a significant difference (ANOVA followed by Tukey-Kramer multiple-comparison test, p <0.05).

We measured the level of PTTH mRNA and PTTH content in the brain of post-diapause pupae to determine what was responsible for the low PTTH titers noted during early post-diapause development. PTTH mRNA levels gradually increased until 36 h after warming and then decreased (Fig 6A). The level of PTTH mRNA at the peak was similar to that in nondiapause pupae. The PTTH content in the brains of post-diapause pupae was around 0.15 U, much less than that in nondiapause day-0 pupae, which was approximately 0.5 U (Fig 6B). The CC-CA of post-diapause pupae also contained little amount of PTTH (0.07, 0.10, 0.12, 0.07 and 0.09 U at 0, 12, 24, 36 and 48 h after warming, respectively). These results suggest that the transcription of the *PTTH* gene is upregulated but its translation is not, causing a relatively low titer of PTTH in the hemolymph.

### The sensitivity of the PG to PTTH in post-diapause pupae

**Fig 7:**
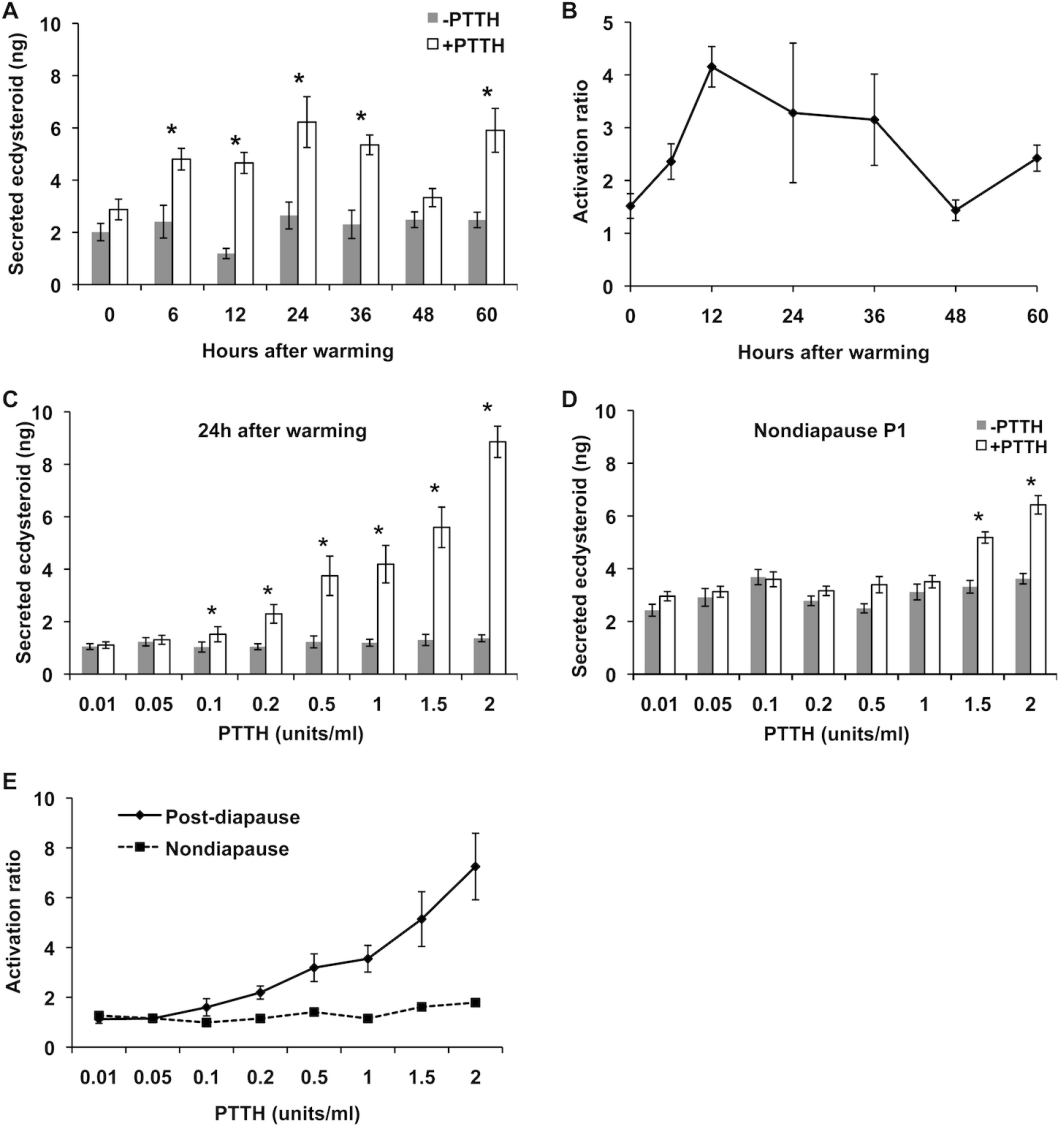
The responsiveness of the PGs to PTTH in post-diapause pupae. (A) The PGs of post-diapause pupae were dissected at various times after warming and incubated *in vitro* with or without PTTH (0.2 units/ml) for 2 h. The amount of ecdysteroid secreted into the medium was determined by ELISA. The values are the means ± SEM (n = 6). (B) Activation ratios were calculated from the data in A. (C, D) The PGs of post-diapause pupae 12 h after warming (C) and of day-1 nondiapause pupae (D) were dissected and incubated *in vitro* with or without PTTH at various concentrations (0.01 to 2 units/ml) for 2 h, and the amount of ecdysteroid secreted into the medium was determined by ELISA. The values are the means ± SEM (n = 8). (E) The responsiveness to PTTH of the PGs of post-diapause pupae and nondiapause pupae was expressed as activation ratio, which was calculated from the data in C and D. *, P < 0.05, Student t-test.

Since the PTTH titer at the beginning of post-diapause development was much lower than expected, we examined whether or not the PGs of post-diapause pupae can be activated by this low titer of PTTH. The PGs were collected from pupae at 0, 6, 12, 24, 36, 48, 60 h after warming, and then ecdysteroid secretory activity of the glands *in vitro* was investigated in the presence or absence of 0.2 U/ml of PTTH that corresponded to the peak titer after warming of pupae. In this experiment, the sensitivity of the PG to PTTH was assessed as the extent to which the secretion of ecdysteroid was enhanced in the presence of PTTH, which is called the “activation ratio” [19]. The sensitivity of the PGs to PTTH was already elevated at 6 h after warming (Fig 7A and 7B). The PGs 6 to 36 h after warming responded well to PTTH, suggesting that the amount of PTTH secreted shortly after warming is sufficient to activate the PGs of post-diapause pupae. This observation prompted us to examine the possibility that the PTTH sensitivity of the PG in post-diapause pupae is higher than that in nondiapause pupae, because the PTTH titer was quite different between post-diapause and nondiapause pupae. Thus, the PTTH sensitivity of the PGs was compared between the post-diapause pupae 24 h after warming and day-1 nondiapause pupae, by changing the concentration of PTTH in the medium. In day-1 nondiapause pupal PGs, the response to PTTH was not observed at PTTH concentrations up to 1.5 U/ml. In contrast, in the post-diapause pupal PGs, the response to PTTH was observed even at a concentration of 0.1 U/ml. With the increase in the PTTH concentration in the medium, the ecdysteroid secretory activity of the PGs increased, exceeding that of day-1 nondiapause pupal PGs at 2U/ml (Fig 7C and 7D). These results indicate that the PTTH sensitivity of the PGs is much higher in post-diapause pupae than day-1 nondiapause pupae.

### *Torso* expression

**Fig 8:**
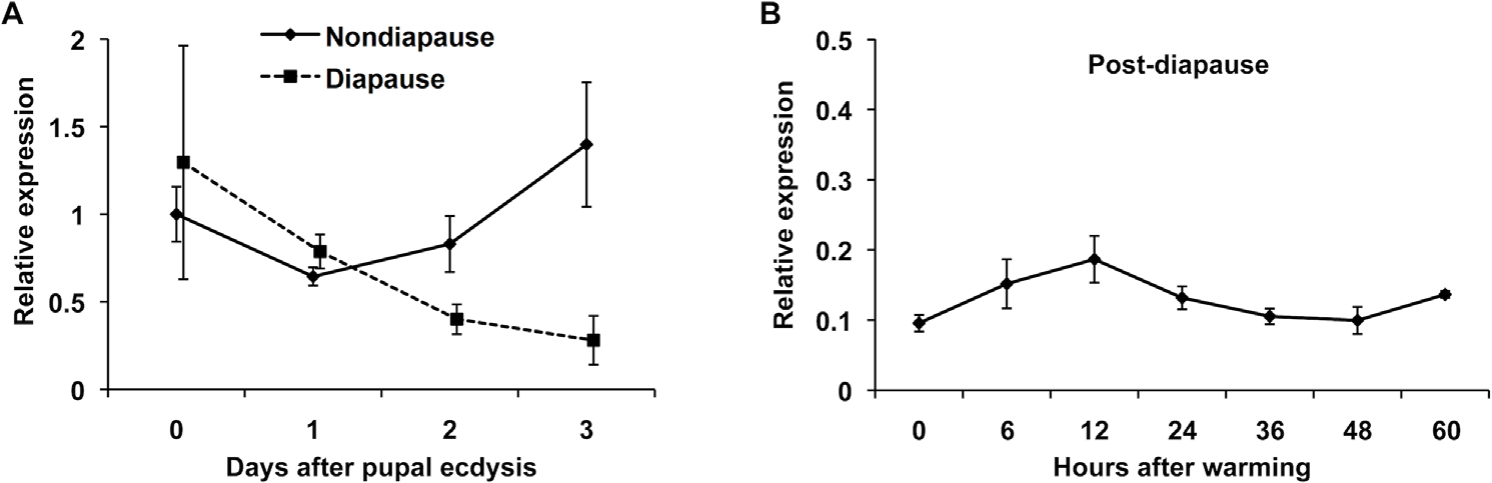
qRT-PCR analysis of *MabTorso* expression in the PGs. The PGs were dissected from nondiapause and diapause pupae 0 to 3 days after pupal ecdysis (A) or from post-diapause pupae at various times after warming (B), and relative expression levels of the *MabTorso* gene were determined by qRT-PCR, with the level in day-0 nondiapause pupae being 1. The values shown are the means (± SEM) of three independent determinations.

Since the sensitivity of the PGs to PTTH in post-diapause pupae is high, we expected that the expression of Torso, a predicted PTTH receptor [20], in these PGs would be higher than those in day-1 nondiapause pupae. To analyze the expression of *Torso*, we first cloned the *M*. *brassicae Torso* gene. The deduced amino acid sequence of the gene showed high homology (72% identity) to *B*. *mori* Torso and contained a highly conserved tyrosine kinase domain (S1 Fig). The expression levels of *Torso* in the PGs during early post-diapause development were measured by qRT-PCR. For comparison, the levels in the PGs of both nondiapause and early diapause pupae 0–3 days after pupal ecdysis were also measured at the same time (Fig 8A and 8B, respectively). While *Torso* expression in nondiapause pupae was maintained at high levels, that in diapause pupae gradually decreased. Unexpectedly, *Torso* expression in the post-diapause pupae was very low. These results indicate that the observed high PTTH sensitivity of the PG in the post-diapause pupae does not result from an elevation in *Torso* expression.

## Discussion

We have clearly demonstrated that PTTH is responsible for the induction of adult development in the post-diapause pupae of *M*. *brassicae*. To our knowledge, this is the first identification of the brain-derived hormone that triggers post-diapause pupa-adult development, in a long history of the study of pupal diapause since the mid 20^th^ century, when Carroll Williams suggested the involvement of a “brain hormone” in this process.

As with pupal diapause in many lepidopteran insects, the diapause in *M*. *brassicae* terminates when the pupae experience a cold temperature for a certain period of time. This period is about 8 weeks in this species (Fig 1), and the 8-week-chilled pupae start adult development if the temperature is raised to a level that allows the animals to develop. It has been well established that a direct trigger of adult development is ecdysteroids secreted by the PGs. However, there has been some debate about what factor activates the PGs to secrete ecdysteroids. In *H*. *cecropia*, the debrained post-diapause pupae never started development, but the implantation of the brain from chilled pupae induced adult development [4]. Based on these observations, Williams hypothesized that diapause terminates when the brain hormone is secreted to activate the PGs. In *Pieris rapae*, however, brain removal before warming of the chilled pupae did not affect the resumption of adult development under warm conditions, suggesting that the PGs had already been activated during the chilling period [21]. Moreover, some insects only need the brain for a relatively short time after entering diapause in order for adult development to resume (for a review, see [22]). Thus, little is known about the mechanisms by which the PGs are activated after diapause termination.

In *M*. *brassicae*, like *H*. *cecropia*, brain removal from post-diapause pupae inhibited adult development, but this inhibition was rescued by brain implantation. Therefore, we decided to identify the factor that is contained in the brain and activates the PGs of post-diapause pupae.

Brain extract successfully induced adult development when injected into debrained post-diapause pupae, and the ability of this extract was abolished when PTTH was specifically removed from the extract by immunoprecipitation with anti-*Mab*PTTH antibody. This result indicates that the factor in the brain extract responsible for the induction of adult development is PTTH. The role of PTTH at this stage was strongly supported by the actual increase in PTTH titer in the hemolymph shortly after warming of the previously chilled pupae. Unexpectedly, the peak titer was rather low as compared to the titers observed during early pupa-adult development of nonodiapause pupae. The low titer of PTTH in the hemolymph was consistent with the small amount of PTTH peptide in the Br-CC-CA. However, this low titer (0.2U/ml) of PTTH is likely still effective to activate the PGs at this stage, because the PGs from the post-diapause pupae responded to a PTTH concentration more than 10 times lower than that which activated the nondiapause pupal PGs.

One possible mechanism to increase the sensitivity of the PGs to PTTH may be via high expression of PTTH receptors in the PG cells. Torso has been identified as a receptor of PTTH in *D*. *melanogaster* [20]. We therefore cloned the *Torso* gene in *M*. *brassicae* and measured the expression level of the gene in the PGs of post-diapause pupae by quantitative RT-PCR. However, *Torso* expression levels were rather low compared to levels in the PGs of nondiapause day-0 pupae. Although a possibility that Torso protein is abundantly expressed in the PG cell membrane cannot be excluded, this observation suggests that the expression level of Torso is not responsible for a high PTTH sensitivity of the PGs at this stage. Another possible mechanism for the enhanced sensitivity of the PGs may be related to changes in signal transduction within the cells. It is well known that stimulation of the PGs by PTTH is mediated by cyclic AMP [14]. It is therefore possible that adenylate cyclase activity is high or phosphodiesterase activity is low in the PG cells of post-diapause pupae. The differences in these and other components of PTTH signal transduction pathway must be examined in the future. The changes, if any, in the pathway might be regulated by other hormone, because some hormones or neuropeptides have been demonstrated to stimulate the PGs in other insects (see below).

We can also assume that a PTTH receptor other than Torso may exist, regulating PG activity, because the identification of Torso as a PTTH receptor in *D*. *melanogaster* does not exclude a possible existence of another PTTH receptor(s) in other insects. In fact, the developmental changes in *Torso* expression in *B*. *mori* [20] do not correlate with those in the hemolymph ecdysteroid titer [23]. A search for a novel PTTH receptor is an important and fascinating research subject in the future.

In connection with a high sensitivity of the PGs, it is interesting to speculate that the PGs of post-diapause pupae may be easily activated by a wide variety of factors that potentially stimulate the PGs. JH, for example, has sometimes been reported to activate the PGs in a specific stage of development [24–26]. Therefore, this hormone may be able to terminate diapause by stimulating the PGs in some insects. In fact, Williams reported that CC-CA implantation into debrained post-diapause pupae led to adult development in *H*. *cecropia* [27]. However, it is unlikely that JH serves as a physiological activator of the PGs at this stage of development, because allatectomy of pupae immediately after warming did not affect their development (Fig 3C). Some other factors including bombyxin (4K-PTTH in older literatures)[28], diapause hormone [29, 30], and orcokinin [31] are also reported to stimulate the PGs in some insects under specific conditions. Therefore, these factors might also induce adult development if injected into the debrained post-diapause pupae, although in our preliminary experiments, bombyxin and diapause hormone of *B*. *mori* had no effect on the development of the pupae (data not shown). It should be noted, however, that even if some exogenous hormone(s) stimulates the PGs of debrained diapause or post-diapause pupae, the physiological meaning of that effect must be carefully evaluated by some experiments which include the measurement of the titers of the hormones of interest in the hemolymph.

It is clear that PTTH plays a pivotal role in the induction of adult development of post-diapause *M*. *brassicae* pupae, but when and how long PTTH is necessary to activate the PGs are unclear. When only the brain was removed from the post-diapause pupae, a single injection of PTTH (brain extract) to these pupae could induce adult development, whereas the same injection could not when the CC-CA was removed along with the brain. This observation suggested that some factor(s) in the CC-CA also contributes to the activation of the PGs. The major hormone present in this complex is JH. However, the involvement of JH in the activation of the PG in this process was positively excluded, as discussed above. Thus, we hypothesized that the PTTH contained in the axon terminals in the CA is slowly released into the hemolymph after brain removal and mildly but continuously stimulates the PGs until the PGs are further stimulated by injected PTTH. To examine this hypothesis, we removed the Br-CC-CA from the previously chilled pupa immediately after warming and then implanted a small piece of PTTH-containing gel into the head of the pupa. About 40% of the gel-implanted pupae developed into the adults, strongly supporting our hypothesis. It is likely that the PGs of the post-diapause pupae are first weakly activated (or potentiated) by a mild and continuous stimulation of PTTH and then fully activated by a stronger stimulation of PTTH, through an increase in the PTTH titer of the hemolymph.

The present study has demonstrated that PTTH is a key factor in initiating post-diapause development in *M*. *brassicae*. However, it is unclear if PTTH is involved in this process in other insects. There are many examples where the brain is not necessary for the post-diapause pupae to resume adult development. However, the eventual adult development of the brainless pupae does not necessarily indicate that PTTH does not contribute to the activation of the PG, because it is possible that PTTH, if provided, may facilitate the activation of the PGs, even if the PGs are eventually activated after a long time without the stimulation by PTTH. Hence, PTTH might be generally responsible for the timely activation of the PGs in the post-diapause pupae. Alternatively, the involvement of PTTH in the initiation of post-diapause development may be limited to some insects, including *M*. *brassicae*. Further studies using many other species of insects are necessary to establish a general role for PTTH in the regulation of post-diapause pupa-adult development.

Our study has revealed that diapause development proceeds in both the brain and the PG under low temperature conditions: the brain acquires the ability to release PTTH and the PG a high responsiveness to PTTH. At present, it is unknown what mechanism(s) is involved in the regulation of diapause development by a low temperature and how these organs acquire such capabilities. To elucidate the mechanism of environmental control of diapause development, it is necessary first to disclose the changes occurring in these organs at the cellular and molecular levels in the chilled diapause pupae. Such studies are now underway.

## Supporting Information

S1 Fig Amino acid sequence of *M*. *brassicae* Torso. The deduced amino acid sequence of *M*. *brassicae* Torso (MbTorso) is shown together with that of *B*. *mori* Torso (BmTorso). Black boxes denote conserved amino acids.
(DOCX)
