# Supplementary material for: Endocrine Mechanisms Regulating Post-Diapause Development in the Cabbage Armyworm, Mamestra brassicae
Source: PLoS One. 2016 Jan 8;11(1):e0146619. doi: 10.1371/journal.pone.0146619 (PMC4706342; doi:10.1371/journal.pone.0146619)
Supplement: S1 Fig — The deduced amino acid sequence of M. brassicae Torso (MbTorso) is shown together with that of B. mori Torso (BmTorso). Black boxes denote conserved amino acids. (DOCX) [file pone.0146619.s001.docx]

MabTorso MFHKKFEHYIIFCLIKQITMILFVCGKPTDSDETKLFTDDQLFDLAESVCYDLEFVIN-V 59

BomTorso MYSE--GKLLKVFLIFAGFIIFSLCGEVVS--QRYPPAPGLLKYLEQDVCYSLYYYLNWT 56

MabTorso PLKTCEASFWAPKTRTAEQLSEVTIKCQTNKSMTFHIDPSESYQLVALVPHVTNDSDTLS 119

BomTorso SLADCKTNF--EETGISDVPSTVKVRCQSKNSIRFETEPSEHWQLFILMEHDNFDP---- 110

MabTorso YINYLVIDPKQDQGVISTTPTRNYTIWRALLEQ-GSPVEWRGGEQAPEYN-EYTVPEHKR 177

BomTorso -IPFTLIEPNNVFGELITTANKEYQIWSTYLDEYGTLQDWMEGPIVLKFDQRNQQPDDIK 169

MabTorso YEIKISQK---PSSNGSSVLASFTWNTTGDCDIWFDVYNMCTNISTKSTKVLQKRRDDEP 234

BomTorso YNVTQEFKYIILGNDSYTINGKFVWNTTGDRDLCFDIANICQNTNMKHAKIWP---TAHP 226

MabTorso HVDFDQPTFDDDCDLEVTGMFGTSHLKYQTHSCKEI-DCTPKEIKPPMPDNITIEAIKDS 293

BomTorso SFDVENLVLNDECEIHVKGIHGTTKHKYKTPSCFELPECFLNNMEPEIPQDVAIAADQDL 286

MabTorso IDTWEVHLNWTLPNRNPDSYKP----------------------------LLCLQYMVTC 325

BomTorso RGWWNINVAWAKPHFQPEIYNVTVRANMIRSIILPGNATETTFRNIPNTFLSAGKIYNVS 346

MabTorso LYYYLGMQQKLCSKKCRVK-----EWRSTTFGCKPDKAYRCLHQQACTIPRNMGGPVPPY 380

BomTorso VYAIIGQKASHTSRRAFTPGMLRWVWAGATAGAGCAAGGLLAATLLCCGHRRATSRVSQE 406

MabTorso KPDDKPSKDSDTEVLEIWS-ETEDRWEVRADKLVLHEVIGEGAFGVVRRATLAPHGVQVA 439

BomTorso DPDEKTPKEDDVEIIGIESGSADDHWEVRSDRVLLHEVIGEGAFGVVRRGTLAPGGKSVA 466

MabTorso VKMLKDFPSLDEIRSFRSEMELMKSVGVHPHLVSLVGCCTGRRPLIVAEYCSRGDLLSYL 499

BomTorso VKMLKEFPSQEEVRSFRSEMELMKSVGAHPHVVSLVGCCSGRKPLIVAEYCSRGDLLSYL 526

MabTorso RCTWDVMLSKRNAKYYNNNIDISDYRNDLFKCKTQMESSKLVVNKLYELQEICDKELTAL 559

BomTorso RSSWDIIVSKHTAKYYNNNMDSMDTS----KLKVHKEHTKLVVNKLYELQGPCETELTPL 582

MabTorso DLLSFCRQIAMGMEFLAANRVVHRDLAARNILVTGDRTLKIADFGLSRDVYEENQYKQKG 619

BomTorso DLLSFCRQIAMGMEFLASNRIVHRDLAARNVLVTEDKTLKIADFGLSRDIYEENQYKQKG 642

MabTorso NGKMPVKWMALESLTRRIYTTQSDVWSFGVVVWEIATVGGAPYASVPGARLPRLLRSGYR 679

BomTorso NGKMPVKWMALESLTRRVYTTQSDVWSFGVVIWEIVTVGGSPYPEVPAARLVRSLRSGYR 702

MabTorso MPKPNNCSPQLYDLMLSCWRTHPRERPTFAELHQRLDELLNSACANQYLSLELDADDAPP 739

BomTorso MPKPVNCSKPLYDIMRACWNASPRDRPTFPELHQKLDDLLHSACANEYITLEVDVDEAPS 762

MabTorso TPKHHRYIKMLMRGKRSWTRGETYERPLKAAQRTNHYTTPPDSLSAHPV 　　　　　 788

BomTorso TPKPQRYIKMLIRGKLPWSR-ESYERPVNPTS--NLYSSPPVIQTKTA- 807

S1 Fig. Amino acid sequence of *M. brassicae* Torso.
